# ﻿A review of the spider genus *Chthonopes* (Araneae, Theridiosomatidae), with descriptions of two new species from China

**DOI:** 10.3897/zookeys.1124.89991

**Published:** 2022-10-17

**Authors:** Weicheng Yang, Hao Yu, Yucheng Lin

**Affiliations:** 1 School of Life Sciences, Guizhou Normal University, Guiyang, Guizhou 550025, China; 2 Key Laboratory of Bio-resources and Eco-environment (Ministry of Education), College of Life Sciences, Sichuan University, Chengdu, Sichuan 610064, China; 3 The Sichuan Key Laboratory for Conservation Biology of Endangered Wildlife, Sichuan University, Chengdu, Sichuan 610064, China

**Keywords:** Araneoidea, Asia, key, revision, theridiosomatid, Yunnan

## Abstract

The genus *Chthonopes* Wunderlich, 2011 is reviewed in this paper. The type species *Chthonopesjaegeri* Wunderlich, 2011 was illustrated based on new material from the type locality and the new distribution records (Bolikhamsay and Ban Kouanphavang Khammouane, Laos). Two new species are described from Yunnan, China: *C.bifidum* Yu & Lin, **sp. nov.** (♂♀) and *C.jimudeng* Yu & Lin, **sp. nov.** (♀). A key is provided for the genus, as well as species diagnoses, and a distribution map for all five species of *Chthonopes*.

## ﻿Introduction

Theridiosomatidae Simon, 1881 is a small-sized spider family with 20 genera and 135 valid species distributed worldwide, with 11 genera and 28 species recorded from China (World Spider Catalog 2022).

The genus *Chthonopes* was originally erected by Wunderlich (2011) based on two species from Laos, and *C.jaegeri* Wunderlich, 2011 was chosen as the genotype. *Chthonopes* is a relatively small theridiosomatid genus that is distributed exclusively in Laos, with only three species described so far: *C.jaegeri* Wunderlich, 2011, *C.cavernicola* Wunderlich, 2011 and *C.thakekensis* Lin, Li & Jäger, 2014 (World Spider Catalog 2022).

While studying material from Yunnan Province, China, we recognized several specimens belonging to Theridiosomatidae. Detailed study of these specimens reveals that they belong to two undescribed species of *Chthonopes*, a genus previously unknown in China. The goals of this paper are 1) to describe the two new species under the names of *C.bifidum* Yu & Lin sp. nov. and *C.jimudeng* Yu & Lin sp. nov.; 2) to re-illustrate *C.jaegeri* based on new material from Laos, and give supplementary micrographs; and 3) to conduct a comprehensive review of the genus *Chthonopes*, including an identification key and a distribution map for all species.

## ﻿Materials and methods

Specimens were examined and measured with a Leica M205 C stereomicroscope. Further details were studied with an Olympus BX43 compound microscope. Male and female copulatory organs were examined after they were dissected and detached from the bodies. Epigyne were removed and treated with lactic acid before photographed. All specimens were preserved in 95% ethanol. Photos were taken with a Canon EOS 60D wide zoom digital camera (8.5 megapixels) mounted on an Olympus BX43 stereomicroscope. The images were montaged using Helicon Focus ver.3.10 (Khmelik et al. 2006) image stacking software. All measurements in the paper are in millimetres. Leg measurements are given in the following sequence: total length (femur, patella, tibia, metatarsus, and tarsus).

The distribution map was generated with ArcGis ver.10.5 (Environmental Systems Research Institute, Inc.). Locality coordinates for all species are copied from the original publications (see Wunderlich 2011; Lin et al. 2014).

Abbreviations used in the text and figures are as follows:

**Asp** accessory spermathecae;

**CD** copulatory duct;

**CL** cymbial lobe;

**Co** conductor;

**DA** distal apophysis on tegulum;

**DH** distal horn on median apophysis;

**ED** embolic distal end;

**Em** embolus;

**FD** fertilization duct;

**MA** median apophysis;

**Pc** paracymbium;

**Sc** scape;

**Sp** spermathecae;

**St** subtegulum;

**Te** tegulum;

**TTr** tibial trichobothium.

All examined materials are deposited in the Natural History Museum of Sichuan University in Chengdu (**NHMSU**), China.

## ﻿Taxonomy

### ﻿Family Theridiosomatidae Simon, 1881

#### ﻿Genus Chthonopes

Taxon classificationAnimaliaAraneaeTheridiosomatidae

Wunderlich, 2011

E038C490-C8DE-5B05-ADB4-3959304A70DC

##### Type species.

*Chthonopesjaegeri* Wunderlich, 2011 from Bolikhansay, Laos, by original designation.

##### Diagnosis.

*Chthonopes* species can be recognised by the copulatory organs: In males, the cymbium apically-ventrally bearing several setae or hairs; median apophysis large and flat, located at the basal or subbasal portion of the tegulum, distally bearing a horn; bulb with an erect distal apophysis located on the apical part of the tegulum; embolus long, accompanied by a tubular conductor, embolic distal end forked. In females, the epigynal plate possesses a scape; vulval center with a V-shaped medial structure; copulatory ducts long, proximally thin but thick-walled, extending anteriorly along flanks of the V-shaped structure, the latter half wide and forming two egg-shaped bursae, surface membranous, wrinkled and ribbed, then connecting with main spermathecae at the central axis of the vulva; main spermathecae small, strongly sclerotized, globular or reniform, separated by about 0.1 – 1.2× their width; hyaline accessory spermathecae located laterally or anterolaterally to main spermathecae, usually claviform or tubular.

##### Description.

See Wunderlich (2011).

##### Composition and distribution.

*Chthonopescavernicola* Wunderlich, 2011 (♂), *C.jaegeri* Wunderlich, 2011 (♂♀) and *C.thakekensis* Lin, Li & Jäger, 2014 (♀) from Laos, *C.bifidum* sp. nov. (♂♀) and *C.jimudeng* sp. nov. (♀) endemic to China.

### ﻿Key to *Chthonopes* species

**Table:** 

1	Males	**2**
–	Females	**4**
2	Anterior eye row with 6 eyes, posterior eye row with 2 eyes; cymbium apically bearing four tiny hairs which are not situated on a hump (Wunderlich 2011: 433, figs 8–10, 16)	***C.cavernicola***
–	Both anterior and posterior eye rows with 4 eyes; cymbium apically-ventrally with a pair of long and bristle-shaped hairs on a hump (Figs 1A, 2C, 3A, 4C)	**3**
3	Distal horn on median apophysis (DH) bifurcate (Fig. 2C); distal apophysis on tegulum (DA) partly membranous or hyaline, whisker-shaped (Fig. 2B, C); paracymbium (Pc) with a spine-like tip (Fig. 2D)	***C.bifidum* sp. nov.**
–	Distal horn on median apophysis (DH) represented by a small needle or spine, not forked, (Fig. 4A–C); distal apophysis on tegulum (DA) relatively sclerotized, lamina-shaped (Fig. 4A–C); paracymbium (Pc) without spine-like tip (Fig. 4D)	***C.jaegeri***
4	Scape (Sc) long, more than 1/2 of epigyne length, rugose (Fig. 3E–G; Lin et al. 2014: 98, figs 17B–E, 18A–C)	**5**
–	Scape (Sc) short, about 1/5 of epigyne length, not rugose (Fig. 1E–G; Fig. 5C–E)	**6**
5	Main spermathecae (Sp) semi-circular, separated by about 1.2× their width; accessory spermathecae (Asp) claviform; fertilization ducts (FD) membranous (Lin et al. 2014: 98, figs 17C, E, 18C)	***C.thakekensis***
–	Main spermathecae (Sp) circular, separated by about 1/3 of their diameter; accessory spermathecae (Asp) consisting of tubular stalk and globular head; FD strongly sclerotized (Fig. 3G)	***C.jaegeri***
6	Scape (Sc) triangular, translucent, extending from posterior margin of epigynal plate; accessory spermathecae (Asp) located anterolaterally to main spermathecae, nearly claviform or tubular (Fig. 1G)	***C.bifidum* sp. nov.**
–	Scape (Sc) digitiform, relatively sclerotized, originating from dorsal side of posterior margin of epigynal plate; accessory spermathecae (Asp) located laterally to main spermathecae, consisting of tubular stalk and globular head (Fig. 5E)	***C.jimudeng* sp. nov.**

#### ﻿ Chthonopes bifidum

Taxon classificationAnimaliaAraneaeTheridiosomatidae

Yu & Lin sp. nov.

3B7D4DB8-4081-585D-BE44-EEE625319199

https://zoobank.org/7F648B7E-A751-4201-B5F4-2F9C2616BDAA

Figs 1 , 2 , 6

##### Type material.

***Holotype***: ♂, China: Yunnan Province: Xishuangbanna: Mengla County: Menglun Town: Shenmi cave, 21.97°N, 101.24°E, elevation 776 m, 3.X.2017, Y. Lin and Y. Li leg. *Paratypes*: 15♀11juv., same data as holotype.

##### Other material examined.

1♂ 24♀ 1juv., China: Yunnan Province: Dehong: Luxi City: Mangliu village: Xianfo cave, 24.33°N, 98.52°E, elevation 1081 m, 25.VIII.2010, C. Wang leg.

##### Etymology.

The species epithet is taken from the Latin adjective “*bifidus*” and refers to the forked distal horn of median apophysis.

##### Diagnosis.

**Figure 1.**
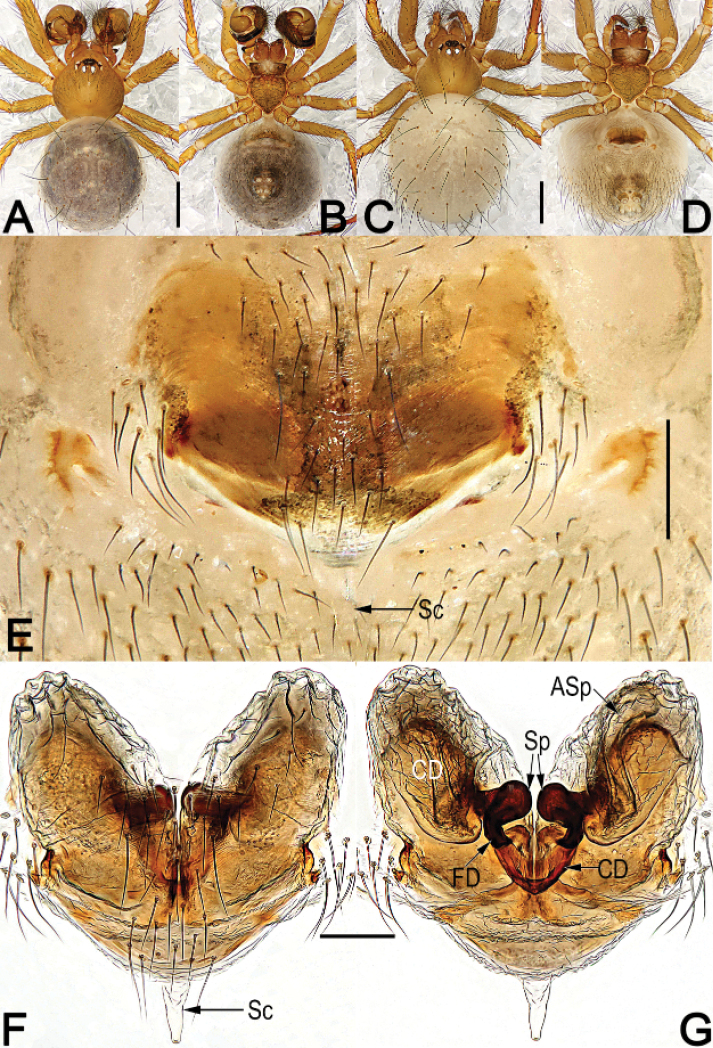
*Chthonopesbifidum* sp. nov., male holotype and female paratype, male habitus (**A, B**), female habitus (**C, D**) and epigyne (**E–G**) **A** dorsal view **B** ventral view **C** dorsal view **D** ventral view **E** intact, ventral view **F** cleared, ventral view **G** cleared, dorsal view. Abbreviations: Asp = accessory spermathecae; CD = copulatory duct; FD = fertilization duct; Sc = scape; SP = spermatheca. Scale bars: 0.5 mm (**A–D**); 0.2 mm (**E, F, G**).

**Figure 2.**
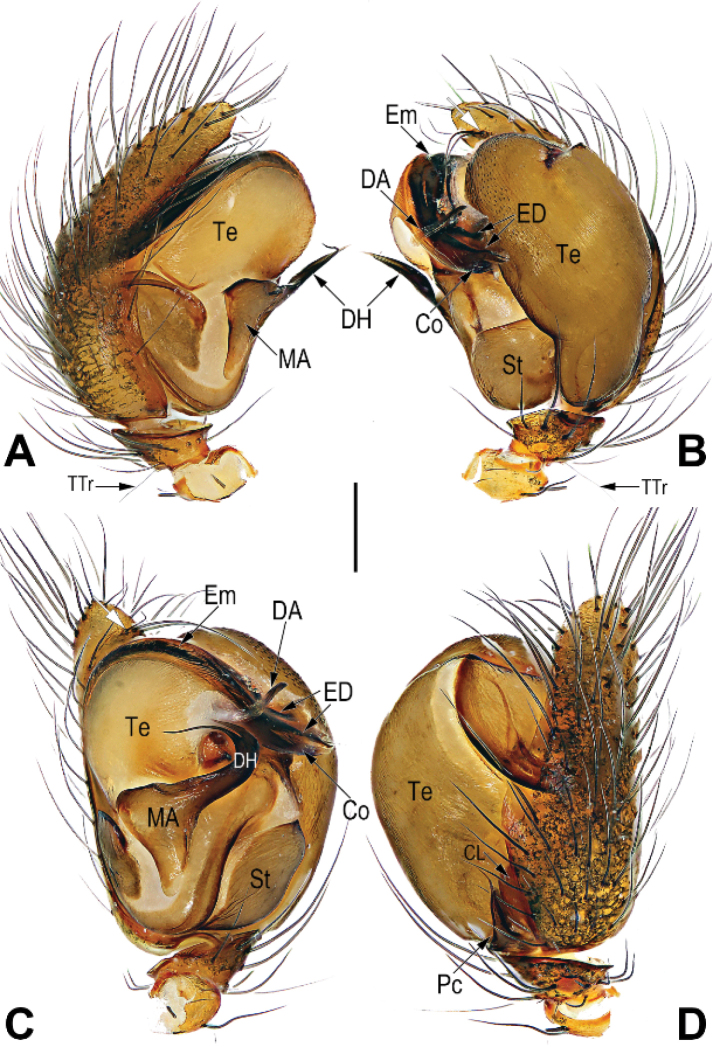
Male palp of the holotype of *Chthonopesbifidum* sp. nov. **A** prolateral view **B** retrolateral view **C** ventral view **D** dorsal view. Abbreviations: Co = conductor; CL = cymbial lobe; DA = distal apophysis on tegulum; DH = distal horn on median apophysis; ED = embolic distal end; Em = embolus; MA = median apophysis; Pc = paracymbium; St = subtegulum; Te = tegulum; TTr = tibial trichobothium. Scale bars: 0.2 mm (**A–D**).

**Figure 3.**
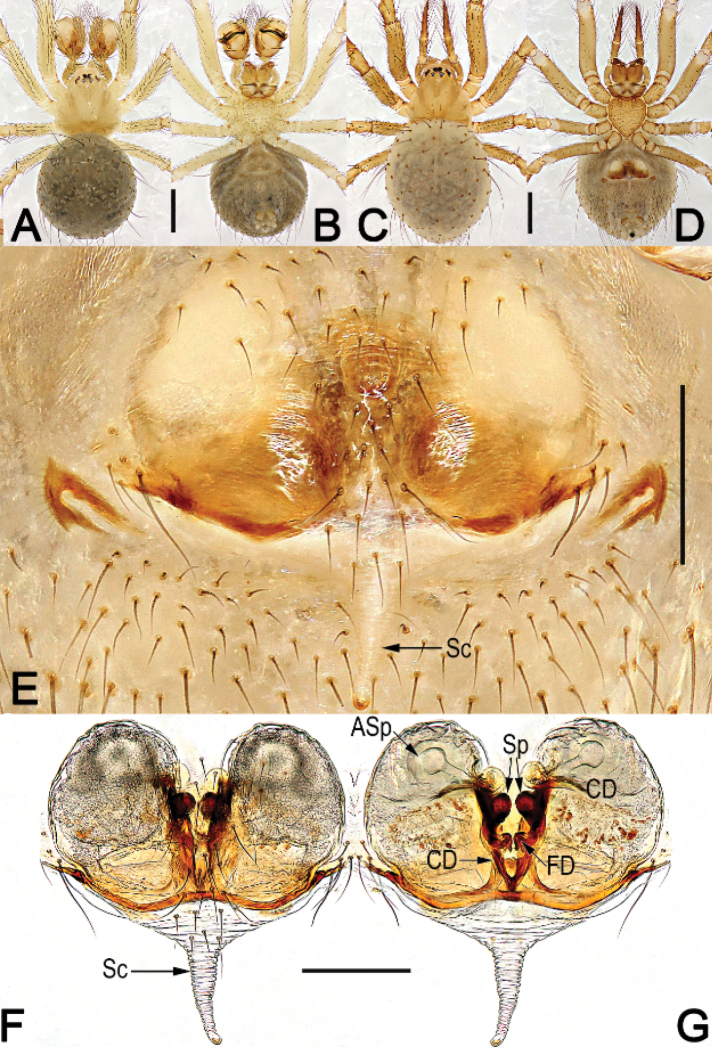
*Chthonopesjaegeri*, male habitus (**A, B**), female habitus (**C, D**) and epigyne (**E–G**) **A** dorsal view **B** ventral view **C** dorsal view **D** ventral view **E** intact, ventral view **F** cleared, ventral view **G** cleared, dorsal view. Abbreviations: Asp = accessory spermathecae; CD = copulatory duct; FD = fertilization duct; Sc = scape; SP = spermatheca. Scale bars: 0.5 mm (**A–D**); 0.2 mm (**E, F, G**).

**Figure 4.**
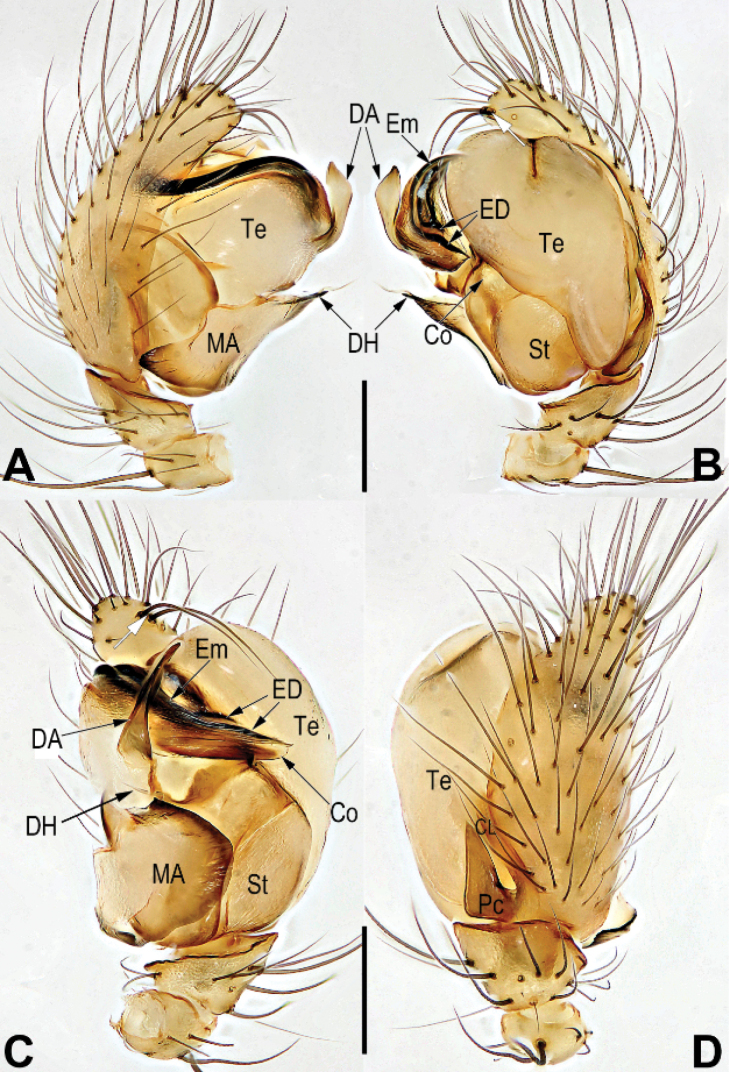
Male palp of *Chthonopesjaegeri***A** prolateral view **B** retrolateral view **C** ventral view **D** dorsal view. Abbreviations: Co = conductor; CL = cymbial lobe; DA = distal apophysis on tegulum; DH = distal horn on median apophysis; ED = embolic distal end; Em = embolus; MA = median apophysis; Pc = paracymbium; St = subtegulum; Te = tegulum. Scale bars: 0.2 mm (**A–D**).

**Figure 5.**
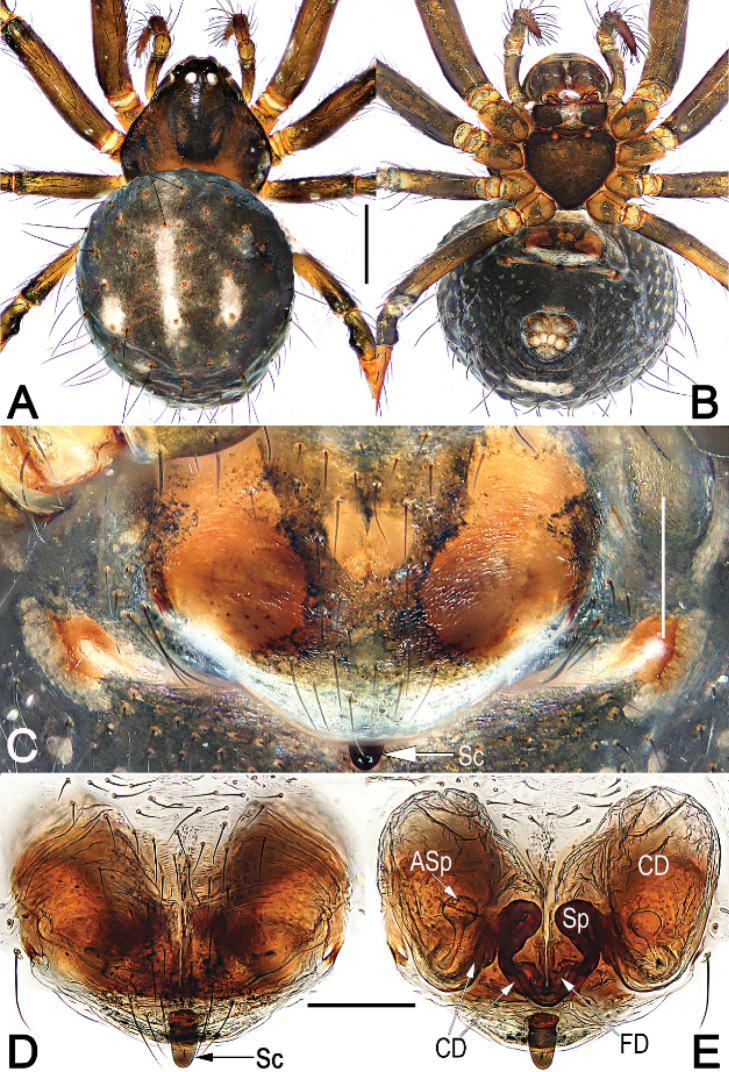
Holotype female of *Chthonopesjimudeng* sp. nov., habitus (**A, B**) and epigyne (**C–E**) **A** dorsal view **B** ventral view **C** intact, ventral view **D** cleared, ventral view **E** cleared, dorsal view. Abbreviations: Asp = accessory spermathecae; CD = copulatory duct; FD = fertilization duct; Sc = scape; SP = spermatheca. Scale bars: 0.5 mm (**A, B**); 0.2 mm (**C, F, G**).

The males of *C.bifidum* sp. nov. easily differentiated from those of all other congeners by the bifurcate distal horn of median apophysis, the partly membranous or hyaline, whisker-shaped distal apophysis of tegulum, and by the paracymbium with a spine-like tip, vs. distal horn on median apophysis represented by a small needle or spine, not forked, distal apophysis on tegulum relatively sclerotized, lamina-shaped, paracymbium without spine-like tip in *C.jaegeri* and *C.cavernicola* (cf. Fig. 2A–D and Wunderlich 2011: 433, figs 13–17, 18b, Fig. 4A–D). The females of *C.bifidum* sp. nov. can be easily distinguished from other congeners except *C.jimudeng* sp. nov. by the short and smooth scape, about 1/5 of epigyne length (Figs 1E–G, 5C–E) (vs. scape rugose, longer than 1/2 of epigyne length in all other congeners, including *C.jaegeri* and *C.thakekensis*; Fig. 3E–G; Lin et al. 2014: figs 17B–E, 18A–C), but differ from the latter by the: (1) scape triangular, translucent, extending from posterior margin of epigynal plate (Fig. 1E–G) (vs. digitiform, relatively sclerotized, originating from dorsal side of posterior margin of epigynal plate; Fig. 5C–E); (2) accessory spermathecae located anterolaterally to main spermathecae, nearly claviform or tubular (Fig. 1G) (vs. located laterally to main spermathecae, consisting of tubular stalk and globular head; Fig. 5E).

##### Description.

**Male** (holotype) (Fig. 1A, B): Carapace nearly pyriform, yellowish brown, without distinct pattern, slightly darker marginally. Anterior eye row recurved, posterior eye row distinctly procurved. Sternum heart-shaped, dark brown, with sparse setae. Mouthparts coloured as sternum. Legs uniformly brown, femora slightly darker. Abdomen round, dorsum centrally beige, marginally coffee coloured with sparse long hairs, weakly ossified at hair base; venter slightly darker than dorsum, posteriorly clothed with short setae. *Measurements*: Total length 2.1. Carapace 0.8 long, 0.9 wide. Clypeus 0.2 high. Sternum 0.5 long, 0.5 wide. Abdomen 1.4 long, 1.3 wide. Length of legs: I 4.0 (1.3, 0.4, 1.0, 0.9, 0.4); II 3.0 (0.9, 0.3, 0.8, 0.6, 0.4); III 2.4 (0.6, 0.2, 0.7, 0.6, 0.3); IV 3.0 (0.9, 0.3, 0.8, 0.7, 0.3).

***Palp*** (Fig. 2A–D): Tibia small, about 1/5–1/6 length of cymbium, dorsally bears a short trichobothium (TTr). Cymbium narrow, about 2.6 × longer than width, with long setae. Paracymbium (Pc) small, about 1/5–1/6 length of cymbium, with a nearly triangular base and spine-like tip. Tegulum (Te) capacious, 1.5 × longer than wide; sperm duct distinct in ventral view, running a V-shaped course along posterior part of the tegulum. Median apophysis (MA) originating from subbasal portion of tegulum, consisting of broad base and biforked distal horn (DH); base nearly triangular; distal horn heavily sclerotized, tip curved and bifurcate, lateral ramus short claw-shaped, mesal ramus filiform and ca. 2 × longer than lateral ramus. Distal apophysis (DA) located at distal-retrolateral position of tegulum, base partly membranous, and tip hyaline with a truncated apex. Embolus (Em) long and thick, hidden behind conductor, arising at approximately the 9–10 o’clock position, terminating at ca. 2 o’clock position, embolic distal end forked. Conductor (Co) tubular, covering almost whole embolus, apex translucent and pointing retrolaterally.

**Female** (one paratype). Somatic features as in Fig. 1C, D and coloration slightly lighter than in male. *Measurements*: Total length 2.5. Carapace 0.8 long, 1.1 wide. Clypeus 0.2 high. Sternum 0.6 long, 0.5 wide. Abdomen 1.9 long, 1.3 wide. Length of legs: I 4.0 (1.4, 0.4, 1.1, 0.7, 0.4); II 3.5 (1.0, 0.4, 1.0, 0.7, 0.4); III 2.6 (0.7, 0.3, 0.7, 0.6, 0.3); IV 3.1 (0.9, 0.3, 0.9, 0.7, 0.4).

***Epigyne*** (Fig. 1C–E). Epigynal plate large, slightly wider than long, with long setae in midline, the arrangement of the various parts of the vulva are indistinctly visible through the tegument; scape (Sc) short, triangular, translucent, extending from posterior margin of epigynal plate, less than 1/5 of epigyne length, apex blunt. The distal part of copulatory ducts (CD) wide, forming two egg-shaped bursae, then connecting with later margin of main spermathecae; the two bursae base closely spaced but anterior surface widely separated by ca. 2× of bursae width. Main spermathecae (Sp) small, reniform, strongly sclerotized, separated by about 1/10 of their width; accessory spermathecae (Asp) located anterolaterally to main spermathecae, translucent, nearly claviform or tubular, about 1/2 of epigyne length. Fertilization ducts (FD), short, ribbon-shaped, strongly sclerotized, located on dorsal-basal surface of main spermathecae; apical parts separated by about 2.5× of FD width, apex curved and sharp.

##### Distribution.

**Figure 6.**
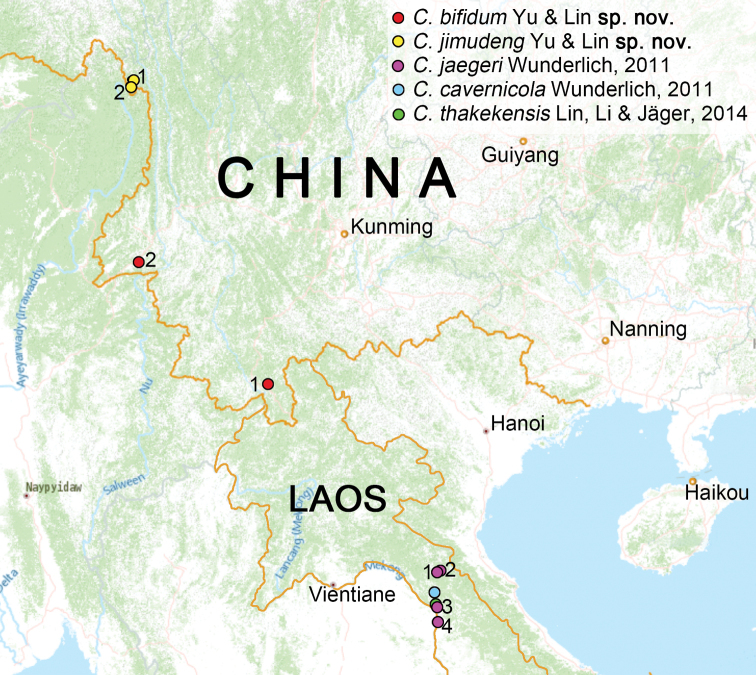
Distribution records of five species of the genus *Chthonopes: C.bifidum* sp. nov. (scarlet circle: 1. China, Yunnan Province, Xishuangbanna, Mengla County, Menglun Town, Shenmi cave; 2. China, Yunnan Province, Dehong, Luxi City, Mangliu village, Xianfo cave); *C.jimudeng* sp. nov. (yellow circle: 1. China, Yunnan Province, Gongshan County, Dulongjiang Town, Jimudeng village; 2. China, Yunnan Province, Gongshan County, Dulongjiang Town, Maku village); *C.jaegeri* Wunderlich, 2011 (carmine circle: 1, 2. Laos, Bolikhansay Province,, Lak Sao, Tham Mang Kone; 3. Laos, Khammouan Province, Thakek area, Ban Phôungam-Mai; 4. Laos, Bolikhamxay Province: Khamkeut area); *C.cavernicola* Wunderlich, 2011 (light blue circle: Laos, Khammouan Province, Tham Boumlou, inside cave); *C.thakekensis* Lin, Li & Jäger, 2014 (green circle: Laos, Khammouan Province, Thakek area, Ban Phôungam-Mai, Tham Phayat, limestone cave).

Known from Mengla County and Luxi City, Yunnan, China (Fig. 6).

#### ﻿ Chthonopes cavernicola

Taxon classificationAnimaliaAraneaeTheridiosomatidae

Wunderlich, 2011

D997D6D3-FF1B-5002-B2EA-73BB44086CDE

Chthonopes cavernicolus Wunderlich, 2011: 433, figs 8–18 (♂).

##### Material examined.

Not examined.

##### Diagnosis.

See diagnosis for *C.jaegeri*.

##### Description.

See Wunderlich (2011).

##### Distribution.

Laos (Fig. 6).

#### ﻿ Chthonopes jaegeri

Taxon classificationAnimaliaAraneaeTheridiosomatidae

Wunderlich, 2011

72F94882-091B-5FC3-9E52-E59C52DEAE46

Figs 3 , 4 , 6

Chthonopes jaegeri Wunderlich, 2011: 435, fig. 18a–f (♂♀).

##### Material examined.

2♀, Laos: Khammouan Province: Thakek area, Ban Phôungam-Mai, 17.55°N, 104.81°E, elevation 495 m, 25.XI.2012, S. Li leg; 7♀, Bolikhamxay Province: Khamkeut area, 18.22°N, 104.81°E, elevation 495 m, 27.XI.2012, Z. Yao leg; 1♂ 2♀, Bolikhamxay Province: Lak Sao, 17.22°N, 104.81°E, elevation 501 m, 3.III.2010, H. Steiner leg.

##### Diagnosis.

The male of *C.jaegeri* resembles those of *C.cavernicola* (Wunderlich, 2011: 433, figs 8–18) in having a large and flat, laminar median apophysis which bears a tiny needle-shaped distal horn (Fig. 4A–C) (vs. median apophysis relatively small, consisting of triangular base and biforked distal horn in *C.bifidum* sp. nov.; Fig. 2C), but differs in the combination of genitalic and somatic characters: distal apophysis of the tegulum is erect, apex relatively sharp, pointing distally (Fig. 4C; Wunderlich 2011: 435, fig. 18b) (vs. curved, apex truncated, pointing proximally; Wunderlich 2011: 433, fig. 17); cymbium apically-ventrally with a pair of long and bristle-shaped hairs on a hump (Fig. 4C; Wunderlich 2011: 435, fig. 18b) (vs. cymbium bearing apically four tiny hairs which are not situated on a hump; Wunderlich 2011: 433, fig. 17); both anterior and posterior eye rows with 4 eyes (Fig. 3A) (vs. anterior eye row with 6 eyes, posterior eye row with 2 eyes; Wunderlich 2011: 433, figs 8–10). Females of *C.jaegeri* are similar to those of *C.thakekensis* (Lin et al. 2014: 98, figs 17A–E, 18A–C) by the epigynal plate with a long, rugose scape, and by the similar configurations of vulva, but they can be differentiated by the circular main spermathecae separated by about 1/3 of their diameter (Fig. 3G) (vs. semi-circular main spermathecae separated by about 1.2 × their width; Lin et al. 2014: 98, figs 17C, E, 18C), the accessory spermathecae consisting of a tubular stalk and globular head (vs. accessory spermathecae claviform, not subdivided; Lin et al. 2014: 98, figs 17C, E, 18C), and by the strongly sclerotized fertilization ducts (Fig. 3G) (vs. membranous FDs; Lin et al. 2014: 98, figs 17C, E, 18C).

##### Description.

See Wunderlich (2011). Habitus as in Fig. 3A–D, male palp as in Fig. 4A–D, epigyne as in Fig. 3E–G.

##### Distribution.

Laos (Fig. 6).

#### ﻿ Chthonopes jimudeng

Taxon classificationAnimaliaAraneaeTheridiosomatidae

Yu & Lin sp. nov.

97AF7146-A756-5622-9E00-BEE1F67A7B01

https://zoobank.org/27320301-272A-497C-B51C-DB133DDCDF20

Fig. 5 , 6

##### Type material.

***Holotype***: ♀, China: Yunnan Province: Gongshan County: Dulongjiang Town: Jimudeng village, 27.79°N, 98.33°E, elevation 1410 m, 15.VIII.2018, Y. Lin and Y. Li leg. ***Paratypes***: 1♀ 2juv., same data as holotype.

##### Other material examined.

6juv., China: Yunnan Province: Gongshan County: Dulongjiang Town: Maku village, 27.68°N, 98.30°E, elevation 1939 m, 14.VIII.2018, Y. Lin and Y. Li leg.

##### Etymology.

This specific name is taken from type locality; noun in apposition.

##### Diagnosis.

The new species is similar to *C.bifidum* sp. nov. (Fig. 1E–G) in the general appearance of the epigyne (also see above diagnosis for *C.bifidum* sp. nov.). From *C.bifidum* sp. nov., the female of *C.jimudeng* sp. nov. can be easily distinguished by the shape of the scape and accessory spermathecae, as well as the arrangement of the various parts of the vulva: (1) scape digitiform, relatively sclerotized (Fig. 5C–E) (vs. triangular, translucent; Fig. 1E–G); and (2) accessory spermathecae located laterally to the main spermathecae, consisting of a tubular stalk and globular head (Fig. 5C–E) (vs. located anterolaterally to the main spermathecae, nearly claviform or tubular; Fig. 1E–G).

##### Description.

**Female** (holotype) (Fig. 5A, B): Carapace nearly triangular, marginally dark, with a dark, wide V-shaped paramedian stripe starting from behind PLE, almost reaching the indistinct cervical groove. Anterior eye row recurved, posterior eye row almost straight in dorsal view. Sternum heart-shaped, centrally dark brown, marginally dark, with sparse setae. Mouthparts coloured as sternum. Legs dark brown, all legs with conspicuous dark annuli in the distal parts of femur, and patella. Abdomen spherical, covered with sparse long setae, setal base sclerotized; dorsum basically black, centrally with three longitudinal white bands, the medial band relatively long, about 1/2 of abdomen length, the bilateral bands short, about 1/2 length of medial band; venter black, without pattern. *Measurements*: total length 2.3. Carapace 1.0 long, 0.9 wide. Clypeus 0.1 high. Sternum 0.4 long, 0.5 wide. Abdomen 1.6 long, 1.2 wide. Length of legs: I 3.1 (1.0, 0.3, 0.9, 0.6, 0.3); II 2.7 (0.9, 0.3, 0.6, 0.6, 0.3); III 1.8 (0.6, 0.2, 0.4, 0.4, 0.2); IV 2.4 (0.7, 0.3, 0.6, 0.5, 0.3).

***Epigyne*** (Fig. 5C–E). Epigynal plate large, about 1.35× wider than long, with long setae in midline, through which spermathecae and copulatory ducts are indistinctly apparent; scape (Sc) distinctly short, about 1/5 of epigyne length, digitiform, relatively sclerotized, originating from dorsal side of posterior margin of epigynal plate, its tip slightly overpasses the posterior margin, apex blunt. The distal part of copulatory ducts (CD) wide, forming two egg-shaped bursae, then connecting with posterolateral surface of main spermathecae; the two bursae widely separated by one width. Main spermathecae (Sp) small, oval, strongly sclerotized, separated by about 1/3 of their diameter; accessory spermathecae (Asp) located laterally to main spermathecae, consisting of tubular stalk and globular head, translucent, about 1/4 of epigyne length. Fertilization ducts (FD) short, ribbon-shaped or lamellar, heavily sclerotized, located on posterior surface of main spermathecae, curved tips separated by about 3.6× FD width.

##### Male.

Unknown.

##### Distribution.

Known only from Gongshan County, Yunnan, China (Fig. 6).

#### ﻿ Chthonopes thakekensis

Taxon classificationAnimaliaAraneaeTheridiosomatidae

Lin, Li & Jäger, 2014

A3A7BB14-9B13-5BA4-9F64-0DFFF3B0035F

Chthonopes thakekensis Lin, Li & Jäger, 2014: 98, figs 17A–E, 18A–C (♀).

##### Material examined.

None.

##### Diagnosis.

See diagnosis for *C.jaegeri*.

##### Description.

See Lin et al. (2014).

##### Distribution.

Laos (Fig. 6).

## Supplementary Material

XML Treatment for Chthonopes

XML Treatment for Chthonopes bifidum

XML Treatment for Chthonopes cavernicola

XML Treatment for Chthonopes jaegeri

XML Treatment for Chthonopes jimudeng

XML Treatment for Chthonopes thakekensis
